# Diabetic ketoacidosis with pneumomediastinum: a case report

**DOI:** 10.4076/1757-1626-2-8095

**Published:** 2009-09-09

**Authors:** Fadi Makdsi, Victor O Kolade

**Affiliations:** Department of Internal Medicine, University of Tennessee College of Medicine975 East Third Street, Box 94, Chattanooga, TN 37403USA

## Abstract

An 18-year-old male with type 1 diabetes mellitus presented to the emergency department after one day of lethargy and vomiting. Physical examination revealed a dehydrated male with tachycardia and Kussmaul’s respiration. There was subcutaneous emphysema in both supraclavicular regions. Chest auscultation revealed a positive Hamman’s sign. Laboratory investigation was significant for metabolic acidosis with venous blood pH 7.08. Plasma glucose was 1438 mg/dl; ketones were present in the urine. Chest X-ray showed subcutaneous emphysema and pneumomediastinum, which resolved spontaneously within 72 hours of initiation of treatment for diabetic ketoacidosis.

Pneumomediastinum is an uncommon complication of diabetic ketoacidosis. Recognizing that severe diabetic ketoacidosis may cause pneumomediastinum allows for expedient management.

## Case presentation

**Figure 1.**
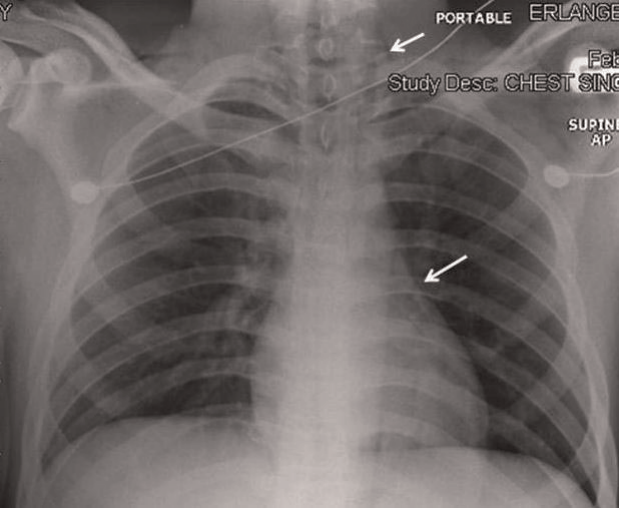
Chest X-ray showing subcutaneous emphysema (upper arrow) and pneumomediastinum at the cardiac border (lower arrow).

**Figure 2.**
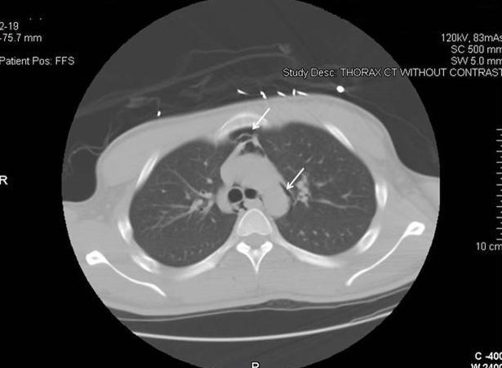
Computed tomography of the chest showing air in the mediastinum, ie pneumomediastinum.

An 18-year-old African-American male with type 1 diabetes mellitus presented to the emergency department with a one-day history of lethargy and vomiting. Clinical examination revealed a dehydrated male with heart rate of 120 beats/min and a blood pressure of 136/66 mmHg. He was tachypneic with a respiratory rate of 35/minute; the pattern was characteristic of Kussmaul’s respiration. There was subcutaneous emphysema in both supraclavicular areas. Auscultation of the chest revealed a crunching noise over the cardiac apex and the left sternal border, synchronous with each cardiac beat (Hamman’s sign). Laboratory investigation revealed a metabolic acidosis with venous blood pH 7.08. Plasma glucose was 1438 mg/dl and bicarbonate < 5 mmol/l; ketones were present in the urine. Chest X-ray showed subcutaneous emphysema as well as pneumomediastinum (Figure 1), confirmed by chest computed tomography (CT) (Figure 2).The subcutaneous emphysema and pneumomediastinum resolved spontaneously within 72 hours of the initiation of treatment for diabetic ketoacidosis (DKA).

## Discussion

Pneumomediastinum (PM) is defined as the presence of gas in the mediastinum. It has been recognized as an uncommon complication of DKA [1]. Vomiting and Kussmaul’s breathing associated with severe DKA can cause alveolar rupture due to increased intra-alveolar pressures with subsequent air leakage along the perivascular sheaths toward the mediastinum. Hamman’s sign is a frequent physical exam finding. A standard chest radiograph or CT can establish the diagnosis of PM. PM in DKA has a relatively benign course, and treatment is mainly supportive. Indeed, the presentation is similar to that of PM occurring spontaneously without a known consequence [2]. Recognizing PM as a cause of severe DKA can prevent unnecessary and expensive investigative procedures by treating physicians.

## Abbreviations

CT: computed tomography
DKA: diabetic ketoacidosis
PM: pneumomediastinum
